# Research note: Protective effects of compound mycotoxin detoxifier in broiler chicken fed aflatoxins-contaminated diets

**DOI:** 10.1016/j.psj.2025.104802

**Published:** 2025-01-10

**Authors:** Kourosh Gholami Ahmadabadi, Mahsa Rahimi, Hamid Raei, Reza Majedifar, Mohammad Amir Karimi Torshizi

**Affiliations:** a Department of Basic Sciences, Faculty of Veterinary Medicine, Science and Research Branch, Islamic Azad University, Tehran, Iran; b Department of Poultry Science, Faculty of Agriculture, Tarbiat Modares University, PO Box 14115-336, Iran

**Keywords:** Aflatoxins, Antioxidant capacity, Broiler, Detoxification, Performance

## Abstract

This study was planned and executed to investigate the effects of two levels of compound toxin binder (**CTB**) on growth performance, serum biochemistry, antioxidant status, intestinal morphology, and the ileal selected microflora population in broiler chickens. A total of 240 one-day-old Ross 308 broiler chickens were divided into four treatments and six replicates (10 chickens per replicate). Experimental groups included; 1, negative control (NC; no aflatoxins (**AFs**) and no additives); 2, positive control (PC; 490 µg/kg AFs); 3, low levels of compound toxin binder (**LCTB**), PC + 1 g/kg available CTB (Navacidox); and 4, high levels of compound toxin binder (**HCTB**), PC + 2 g/kg Navacidox. Increasing body weight gain (**BWG**) following feeding HCTB were found compared to the PC (*P* < 0.05). The highest feed intake (**FI**) was observed in the NC (*P* < 0.05). Feeding broilers with AFs decreased villus height, villus width, and crypt depth compared to the NC (*P* < 0.05). The increasing lactic acid bacteria population in HCTB was recorded compared to the PC (*P* < 0.05). Among biochemistry parameters, total protein, LDL, and uric acid decreased in LCTB compared to the PC (*P* < 0.05). Lower concentrations of ALP, LDH, AST, and ALT in LCTB were observed compared to the PC (*P* < 0.05). Although total antioxidant capacity, GPx, and SOD activities were not affected by treatments (*P* > 0.05), the lowest catalase levels were found in HCTB (*P* < 0.05). In conclusion, AFs feeding impaired growth performance, biochemistry parameters, antioxidant capacity, and intestinal morphology. However, the inclusion of CTB in AFs-contaminated diets effectively mitigated the harmful effects of AFs.

## Introduction

In 1993, the World Health Organization (**WHO**) introduced Aflatoxin (**AFs**) as a group I carcinogen (Karimi Torshizi and Sedaghat, 2023). In addition to the negative impacts of AFs on poultry, these toxins may enter the food chain through transfer from contaminated feed to edible by-products such as liver and eggs (Karimi Torshizi and Sedaghat, 2023). In the past decades, various techniques such as physical, chemical, and biological methods have been developed to find the best approach for removing AFs from feeds (Raei et al., 2022). Based on previous reports, neither of the detoxification methods is fully effective in mitigating the negative effects of mycotoxins (Guo et al., 2023; Karimi Torshizi and Sedaghat, 2023). Therefore, it seems that integrating different methods can intensify the detoxification effect. Physical adsorbents such as montmorillonite and activated carbon, or biological methods such as dietary inclusion of yeast cell wall and probiotics can alleviate the toxic effects of AFs (Liu et al., 2020). Microorganisms such as Lactobacillus, yeast, and Bacillus, or some enzymes such as laccase, can effectively degrade mycotoxin (Liu et al., 2020). Probiotics have been used to prevent intestinal pathogens, improve immune function, and ameliorate the oxidative stress caused by mycotoxins (Karimi Torshizi and Sedaghat, 2023). While several studies have indicated the efficacy of detoxifying compounds, little data have been reported about the dietary inclusion of other compounds, such as organic acids, into routine toxin binders. Hence, the current study investigated the protective effects of a newly developed compound toxin binder (**CTB**) at two levels in broiler chickens' diets contaminated with AFs on growth performance, serum biochemistry, antioxidant status, intestinal morphology, and selected ileal microflora population.

## Materials and methods

### In vitro aflatoxin production

To produce AFs, standard vials of *Aspergillus parasiticus* PTCC-5286 were obtained from the Iranian Research Organization for Science and Technology. *A. parasiticus* was cultured on a yeast extract medium, and after propagation of the fungus, AFs were produced through fermentation on rice.

### Experimental design, birds, and diet

A total of 240 one-day-old broiler chickens (Ross 308) were purchased from a local commercial hatchery. The research was conducted in a completely randomized design with four treatments and six replicates (10 chicks per replicate). Experimental treatments included; 1, negative control (**NC**, no AFs and no additives); 2, positive control (**PC**, 490 µg/kg); 3, low levels of compound toxin binder (**LCTB**), PC + 1 g/kg Navacidox™; and 4, high levels of compound toxin binder (**HCTB**), PC + 2 g/kg Navacidox™. AFs were included aflatoxin B1 = 390 µg/kg, aflatoxin B2 = 10 µg/kg, aflatoxin G1 = 86 µg/kg, aflatoxin G2 = 4 µg/kg. Navacidox™ as an available commercial toxin binder was obtained from Navanik Arman Saba Co., Tehran, Iran, which included heat-processed sodium bentonite, activated carbon, yeast cell wall, organic acids (formic, citric, acetic, and butyric), and *Bacillus subtilis* with a concentration of 10^12^ cfu/kg. Broilers were reared under an environmentally controlled room in wire pens equipped with a drinker. Birds were free access to water and feed throughout the experiment. The house temperature was set at 33°C during the first three days and then gradually reduced until reaching to 24°C. The lighting program was adjusted to 20 h light: 4 h dark through 40 d.

### Growth performance

Growth performance parameters including body weight gain (**BWG**), feed intake (**FI**), and feed conversion ratio (**FCR**) were calculated weekly throughout the experiment. Birds were monitored for general signs such as alertness, fecal consistency, and feather shine twice daily. Mortality in each pen was recorded daily throughout the experiment. Body weight and FI were recorded daily and presented as 2-40 d.

### Serum biochemical assay

At 40 d of the experiment, 2 birds from each replicate were selected and blood samples were taken from the brachial vein. After centrifuging blood samples at 1500 × g for 10 min at 40 d, serum was collected and stored at −20°C until further analysis. The levels of lactate dehydrogenase (**LDH**), aspartate transaminase (**AST**), alanine transaminase (**ALT**), gamma-glutamyl transferase (**GGT**), alkaline phosphatase (**ALP**), glucose, total protein, albumin, uric acid, low-density lipoprotein cholesterol (**LDL**), and high-density lipoprotein cholesterol (**HDL**) were measured with a spectrophotometric method using commercial kits (Pars Azmun, Iran) and a microplate reader (Stat Fax 3200; Awareness Technology Inc., Palm City, FL) as per manufacturers’ instructions.

### Antioxidant parameters

To evaluate of antioxidant status of broilers at 40 d, blood plasma total antioxidant capacity, the activity of glutathione peroxidase (**GPx**), catalase (**CAT**), and superoxide dismutase (**SOD**) were measured using colorimetric methods with a spectrophotometer (BioTec Epoch; BioTec, Winooski, Vermont, USA). In the blood plasma, the total antioxidant capacity, the activity of GPx, CAT, and SOD was assayed using commercial kits (Teb Pazhouhan Razi, Tehran, Iran) according to the guide provided by the manufacturer.

### Jejunal morphometric analysis

After blood collection, selected chickens were humanely euthanized. Jejunal of experimental chicks were sampled and fixed in 10 % neutral buffered formalin for 48 h. Fixed samples were processed with increasing concentrations of alcohol (70, 80, 90, 95, and 100 %) and xylene and then embedded in paraffin. The samples were cut to a thickness of 6 μm using a rotary-type microtome and were stained with hematoxylin and eosin (HE staining). Slides were evaluated to measure villus height (**VH**), villus width (**VW**), the depth of the crypts (**CD**), number of goblet cells, and villus height to crypt depth ratio (**VH:CD**) using a compound Olympus-BX50 light microscope (Olympus Optical) at 40 × and 400 × magnifications.

### Selected ileal microflora population

To assess the selected ileal microflora, ileal digesta samples were collected aseptically on day 40. In summary, a series of dilutions were prepared from 1.00 g of homogenized ileal content, ranging from 10 ^to 1^ to 10^-8^ in phosphate-buffered saline (0.1 M, pH 7.0). Subsequently, 0.1 mL samples from the final three dilutions were plated onto MacConkey agar, MRS agar, and Plate Count Agar (Merck, Darmstadt, Germany) to quantify *E. coli*, lactic acid bacteria, and total aerobic bacteria, respectively. Each dilution was inoculated in duplicate on the respective media and incubated at 37°C for 24 h for enumeration. The resulting data were reported as log_10_ colony-forming units (**CFU**) per gram of ileal content.

### Statistical Analyses

Shapiro–Wilk and Levene's tests were used for normality and homogeneity of variance tests, respectively. After one-way analysis of variance (**ANOVA**) by SAS software, statistical differences among groups were determined by Tukey test. The significance level was set at *P* < 0.05.

## Results and discussion

As indicated in Table 1, broiler feeding with AFs did significantly reduce BWG (−29.6 %) in comparison with the NC (*P* < 0.05). The inclusion of HCTB in the PC diets increased BWG by about 13.7 % (1961 vs 1692 g; *P* < 0.05). The highest FI was observed in the NC (4121 g; *P* < 0.05). Although FI was not significantly different between the PC and CTB groups (*P* > 0.05), adding HCTB numerically increased FI compared to the PC (3549 vs 3314 g). The highest FCR was recorded in the PC (1.96; *P* < 0.05). The LCTB group's FCR was 7.6 % lower than that of the PC (1.81 vs. 1.96). Various studies have confirmed the harmful effects of AFs on BWG, FI, and FCR in broiler chickens (Guo et al., 2023; Raei et al., 2022; Malekinezhad et al., 2021). The reduction in BWG during AF exposure in broilers is linked to the impairment of gastrointestinal tract function, which manifests as decreased protein synthesis, nutrient malabsorption, and impaired production and secretion of digestive enzymes (Malekinezhad et al., 2021). The reduction in the activity of pancreatic enzymes, such as amylase and trypsin, which are crucial for digestion, leads to decreased protein digestibility and nutrient availability. This decline, because of feeding AF-contaminated diets, ultimately compromises poultry performance (Malekinezhad et al., 2021). Since the small intestine is the first organ of AF contact with the bird's body, physically reducing or removing it can ameliorate its harmful impacts on the digestive system (Guo et al., 2023). In the current study, the physical components of the present toxin binder, including bentonite and activated charcoal, through the adsorption of AFs from the intestinal surface might improve growth parameters. Montmorillonite, as a major component of bentonite, inhibits the absorption of AF through its interlayer. The planarity of the AFB1 ring, via the chemisorptive mechanism with high enthalpy, helps its adsorption by the interlayer of montmorillonite (Guo et al., 2023).

**Table 1:** Growth performance, jejunal morphometric parameters, and selected ileal microbiota of broilers fed aflatoxins contaminated diets with various levels of compound toxin binder (**CTB**).

Item	NC^1^	PC^1^	PC + LCTB^1^	PC + HCTB^1^	SEM	*P*-value
**Growth performance**						
Body weight gain (g), 2-40 d	2405^a^	1692^c^	1882^bc^	1961^b^	30.21	<0.0001
Feed intake (g), 2-40 d	4121^a^	3314^b^	3467^b^	3549^b^	70.53	<0.0001
Feed conversion ratio, 2-40 d	1.71^b^	1.96^a^	1.81^ab^	1.84^ab^	0.0002	0.0025
**Jejunum morphometric parameters**						
Villus height (µm)	1014^a^	833^b^	817^b^	888^b^	21.35	<0.0001
Villus width (µm)	98^a^	70^c^	73^bc^	79^b^	10.29	<0.0001
Crypt depth (µm)	276^a^	184^b^	179^b^	168^b^	10.29	<0.0001
Villus height: crypt depth	3.68^b^	4.62^ab^	4.68^ab^	5.30^a^	0.27	0.005
S**elected ileal microbiota (Log CFU/g)**						
*E-coli*	8.47^b^	8.93^a^	8.81^ab^	8.73^ab^	0.06	0.003
Lactic acid bacteria	8.88^a^	8.46^c^	8.64^ab^	8.84^bc^	0.03	<0.0001
Total aerobic bacteria	8.45^b^	8.50^a^	8.55^a^	8.28^ab^	0.02	0.06

1 NC, negative control; PC, positive control; LCTB, low level of compound toxin binder; HCTB, high level of compound toxin binder.

a-c Values within the same row followed by different superscript letters differ significantly (*P* ≤ 0.05).

As shown in Table 1, the highest VH, VW, and CD significantly were measured in the NC (*P* < 0.05). Although there was no significant difference between the PC and CTB groups (*P* < 0.05), the VH (+6 %) and VW (+11 %) in the HCTB were numerically higher than in the PC. Compared to the NC, VH:CD significantly increased in the HCTB (5.30 vs 3.68; *P* < 0.05). The small intestine is a vital section due to its role in digestion and absorption of nutrients. The major toxic effects of AFs include the loss of barrier function, the induction of inflammatory responses due to the infiltration of white blood cells, structural damage characterized by mucosal hyperplasia, and vacuolar degeneration. These changes are also associated with a reduction in VH and an increase in CD (Hernández-Ramírez et al., 2020). Toxin binders including probiotics such as Bacillus subtilis break down the AFB1 molecular structure through a fermentation process. This process creates a favorable environment in the gastrointestinal system, ultimately improving intestinal morphology and integrity (Guo et al., 2023). Probiotics, organic acids, and yeast cell wall, as components of the present toxin deactivators, have beneficial impacts on intestinal morphology by improving and maintaining a healthy gut structure in broilers (Karimi Torshizi and Sedaghat, 2023; Kyoung et al., 2023; Ebeid et al., 2021). Mannan oligosaccharides and β-glucan in the yeast cell wall are instrumental in maintaining intestinal health by preventing pathogens from binding to the villi (Kyoung et al., 2023).

The population of *E. coli* (+5 %) and total aerobic bacteria (+4 %) increased in the PC compared to the NC (Table 1; *P* < 0.05). There were no significant differences in *E. coli* and total aerobic bacteria populations between the NC and HCTB. A higher population of lactic acid bacteria in the HCTB was observed compared to the PC (*P* < 0.05). A reduction in the *E. coli* population and an increase in the lactic acid bacteria population following the dietary inclusion of compounds containing probiotics, organic acids, and yeast cell walls have been reported in previous studies (Karimi Torshizi and Sedaghat, 2023; Kyoung et al., 2023; Guo et al., 2023). Probiotics play a crucial role in diminishing the population of harmful pathogens through multiple pathways, including the competitive exclusion of pathogens, competition for nutrients, reinforcement of the epithelial barrier, synthesis of numerous antimicrobial peptides, and promotion of immune modulation in the gut. Mannan oligosaccharides have a good potential to attach pathogens, which confirms yeast cell wall to enable positive alterations in ileal microbiota composition (Kyoung et al., 2023). Organic acids such as formic, citric, and butyric inhibit the growth of harmful bacteria by influencing the pH (Pearlin et al., 2020).

Although, glucose and HDL were not significantly affected by treatments (Table 2; *P* > 0.05), total protein and LDL in LCTB decreased compared to NC (*P* < 0.05). Albumin levels were not significantly different between the NC and CTB groups. Lower uric acid concentrations were observed in LCTB than in the NC and HCTB (*P* < 0.05). Excluding GGT, the activity of other enzymes was significantly affected by experimental diets (*P* < 0.05). The PC exhibited the highest levels of ALP, LDH, AST, and ALT compared to other groups (*P* < 0.05). Compared to PC, the activity of ALP, LDH, AST, and ALT was lower in LCTB (*P* < 0.05). The highest total antioxidant value was observed in HCTB, although, this change was not significant (*P* > 0.05). The PC had higher catalase activity than the LCTB (1.34 vs 0.68 mU/mL; *P* < 0.05). However, this parameter was not significantly different between PC and LCTB (1.18 vs 1.34 mU/mL; *P* > 0.05). Many studies have shown that animals fed AFs-contaminated diets in a range of 40 ppb to 2 ppm exhibited elevated liver enzyme activity (Karimi Torshizi and Sedaghat, 2023; Raei et al., 2022; Malekinezhad et al., 2021). The elevation in the activities of ALT, AST, LDH, GGT, and ALP following such exposure indicates possible disruption of liver cells, likely due to necrosis or alterations in membrane permeability (Raei et al., 2022). The decrease in the activity of ALP, LDH, AST, and ALT enzymes in the CTB groups compared to the PC indicates mitigating the toxic effects of AFs on the liver. On the other hand, liver injury by impairing the normal metabolism of nutrients changes biochemistry characteristics. The presence of mycotoxins such as AFB1 diminishes the glomerular filtration capacity, leading to a decrease in serum glucose, total protein, and albumin levels, alongside an elevation in serum urea, creatinine, and ammonia concentrations (Guo et al., 2023; Raei et al., 2022). Mycotoxin consumption reduces the antioxidant capacity of birds through a negative impact on antioxidant parameters such as GPx, SOD, and CAT activity (Chen et al., 2022). AFs, especially AFB1, cause the accumulation of reactive oxygen species to exceed the concentration of antioxidant parameters leading to lipid peroxidation (Raei et al., 2022). AFB1 through impairing intestine function including digestion and absorption of vital nutrients such as fats and vitamins including C, E, and A may disrupt normal antioxidant performance (Malekinezhad et al., 2021).

**Table 2:** Serum characteristics, enzymes activities, and antioxidant status of broilers fed aflatoxins contaminated diets with various levels of compound toxin binder (**CTB**).

Item^2^	NC^1^	PC^1^	PC + LCTB^1^	PC + HCTB^1^	SEM	*P*-value
**Serum biochemistry**						
GLU (mg/dL)	337^ab^	364^a^	328^b^	326^b^	6.66	0.05
TP (g/dL)	4.68^a^	4.19^ab^	3.95^b^	3.99^b^	0.15	0.01
ALB (g/dL)	3.50^a^	2.76^b^	3.07^ab^	2.98^ab^	0.14	0.01
HDL (mg/dL)	46.3^a^	37.0^ab^	31.7^b^	39.2^ab^	3.48	0.05
LDL (mg/dL)	161^a^	126^ab^	94^b^	113^ab^	10.98	0.003
UA (mg/dL)	4.62^a^	3.83^ab^	2.96^b^	4.36^a^	9.93	0.05
**Enzymes activities**						
ALP (U/L)	380^b^	490^a^	385^b^	413^ab^	14.40	0.0021
LDH (U/L)	782^b^	1161^a^	887^b^	741^b^	27.23	<0.0001
GGT (U/L)	29.8	36.1	17.7	26.6	2.42	0.09
AST (U/L)	112^b^	145^a^	105^b^	119^ab^	4.90	0.0021
ALT (U/L)	9.8^b^	17.1^a^	13.0^b^	11.0^b^	0.53	<0.0001
**Antioxidant status**						
Total antioxidant (U/mL)	1.52	1.64	1.52	1.70	0.09	0.45
GPx (mU/mL)	0.170	0.171	0.178	0.174	0.003	0.32
CAT (mU/mL)	1.28^ab^	1.34^a^	1.18^ab^	0.68^b^	0.03	0.03
SOD (U/mL)	1.16	1.03	1.17	1.08	0.09	0.24

1 NC, negative control; PC, positive control; LCTB, low level of compound toxin binder; HCTB, high level of compound toxin binder.

2 GLU, glucose; TP, total protein; ALB, albumin; HDL, high-density lipoprotein cholesterol; LDL, low-density lipoprotein cholesterol; UA, uric acid; ALP, alkaline phosphatase; LDH, lactate dehydrogenase; GGT, gamma-glutamyl transferase; AST, aspartate transaminase; ALT, alanine transaminase; GPx, glutathione peroxidase; CAT, catalase; and SOD, superoxide dismutase.

a-c Values within the same row followed by different superscript letters differ significantly (*P* ≤ 0.05).

## Disclosures

The authors declare no conflicts of interest.
